# Supplementary material for: Stigma experiences, effects and coping among individuals affected by Buruli ulcer and yaws in Ghana
Source: PLoS Negl Trop Dis. 2024 Apr 29;18(4):e0012093. doi: 10.1371/journal.pntd.0012093 (PMC11081504; doi:10.1371/journal.pntd.0012093)
Supplement: S2 Material — (PDF) [file pntd.0012093.s002.pdf]

Individual In-depth Interview (IDI) Guide (BU AND YAWS PATIENTS/ PAST PATIENTS)

The researcher is affiliated to the Kwame Nkrumah University of Science and Technology, Ghana. This interview guide is to gather data on your knowledge or thought on the topic: **Stigma experiences, effects and coping among individuals affected by buruli ulcer and yaws in Ghana**. *Your thoughtful and truthful responses will be greatly appreciated. Please answer each question to the best of your knowledge. Your name is not required. The sole purpose of the research is for an academic exercise and your responses will be kept strictly confidential. Thank you for taking time to complete this questionnaire.*

Target Group:
Patients With Skin Condition
Old Patient with Skin Condition (Healed) For Less Than 2 Years

Key Topics:
Internalized Stigma
(Enacted Stigma Experience)
Internalized Stigma
Health Worker Stigma
Effects of Stigma
Coping Strategies to Manage Stigma

**Date (dd/mm/yyyy)**	
**Participant Identifier**	
**Age**	
**Sex**	
**Occupation**	
**Marital Status**	
**Religion**	
**Name of community**	
**Disease characteristics and History of care**	

	**Questions**

General question about patients	Where do you live? Any changes after the disease? Why?<br><br>What did you first do about the disease and why?
Enacted stigma experience	✓ How do people treat you? Friends? Family? Community members? Any difference before, during and after the disease?<br>✓ Are you able go to social gatherings? Market? School? Work? Any changes?<br>✓ Are you able to lead? Any difference before, during and after disease experience? Why?<br><br>✓ How do people treat your family and friends? any changes? specific examples?
Internalized stigma	✓ How do you feel when people get to know about your condition?<br>✓ How do you feel about the way people treat you?<br>✓ What has your sociable life been before and after the disease? Any changes?<br>✓ Did you stay more indoors or outdoors now? Why?<br>✓ Did you feel the need to cover up the lesion from the view of other people? Reasons? Any differences with changes in your social setting?
Health worker stigma	✓ How were you treated by health care care-givers? Any changes?
Effects of stigma	✓ How did the experience affect your life? Social life? work? Mental health? Mood? Academic pursuit?<br><br>✓ How did you handle this disease since it came up? When did you seek care? Why did you choose that pathway?<br><br>✓ Suicidal ideation?<br><br>✓ Were you happy with the way you were treated by people? any specific examples?

Coping strategies	✓ How do you deal with the way you are treated on daily basis? Concealment? substance abuse? Alienation?<br>✓ How has your dressing been like since the onset of the disease? Any differences? Why?
Any additional insights	✓ Do you have further insights you would want to share with us?
