# Supplementary material for: Stigma experiences, effects and coping among individuals affected by Buruli ulcer and yaws in Ghana
Source: PLoS Negl Trop Dis. 2024 Apr 29;18(4):e0012093. doi: 10.1371/journal.pntd.0012093 (PMC11081504; doi:10.1371/journal.pntd.0012093)
Supplement: Attachment — Submitted filename: Response to review comments.docx [file pntd.0012093.s004.docx]

19^th^ March 2024

Kumasi Centre for Collaborative Research

Kwame Nkrumah University of Science and Technology

Kumasi, Ghana

The Editor

PLOS Neglected Tropical Diseases

Dear Sir,

**Re: Decision on your PLOS Neglected Tropical Diseases submission (PNTD-D-23-00867R1) - [EMID:7f58147f870feb51]: ‘Stigma experiences, effects and coping among individuals affected by Buruli ulcer and yaws in Ghana’**

My co-authors and I, wish to thank the editors and reviewers for the feedback on our manuscript.

My co-authors and I have taken note of the review comments and revised the manuscript as suggested. We wish to submit the revised manuscript for publication in your esteemed journal. In the attached reply, the reviewer questions are in blue font and our responses are in red font.

We look forward to the subsequent publication of same.

We count on your usual kind assurances.

Yours Sincerely,

Dr Yaw Ampem Amoako

Corresponding author

**Point-by-point response to review comments**

Reviewer #4: - The objectives of the study are clearly articulated, although there are gaps in the introduction.

- No major remarks to add regarding the study design, population description, sample size or statistical analysis.

- Ethical concerns are present. Please refer to the point-by-point discussion.

Response: we thank the reviewer for making time to provide a review of this manuscript. A detailed point-by-point response is provided below.

Point-by-point:

-    Lines 124-125: “Importantly, there are very few data on how skin NTD stigma affects healthcare- seeking.” Authors are ignoring several pieces of literature, for instance, https://doi.org/10.4269/ajtmh.2002.67.207, https://doi.org/10.1016/j.trstmh.2008.05.026, https://doi.org/10.1093/inthealth/ihv071, doi: 10.1371/journal.pntd.0000237, https://doi.org/10.1371/journal.pntd.0008030, etc.

Response: We have revised the section, and it now reads (line 134-136):

‘’There is some data on how stigma may affect health seeking in NTDs [18, 22-24]. While stigma was reported to delay care seeking in BU [18], Mulder et al [24] did not find a significant relationship between the stigma and delayed presentation.’’

-    Lines 129-132: authors state that "there has been little research on how programs should address the variation or overlap in stigma experiences for different co-endemic skin NTDs", but this formulation raises several questions:

o    It does not necessarily match their objective or even the discussion of their results, as they only briefly mention the practical implications of their results.

o    "varies between conditions and cultures." Was this at any point addressed? If not, why do the authors want to raise this issue?

o    Also, taking as an example, https://doi.org/10.1371/journal.pntd.0008030. Maybe it would be worth revisiting the literature?

Response: We have revised the section, and it now reads (line 140-145):

‘’While some research has been conducted to explore the acceptability and patient experiences of integrated care [27], minimal attention has been made to exploring the overlap of stigma in co-endemic NTDs. This evidence may be useful in informing programs about the stigma overlaps and nuances between co-endemic skin NTDs and the general and specialized support that can be provided to individuals affected with different diseases.’’

-    Lines 132-136: if integration of stigma toward NTD’s was the goal, wouldn't a systematic review of the literature serve that purpose better? If note, why not select also/instead patients with Leprosy or Leishmaniosis? What would you expect to change in your results?

Response: Thank you for you for your comment.

While leprosy has been reported country wide and leishmaniasis has been reported from communities in southeastern Ghana (see Akuffo RA, et al. Endemic infectious cutaneous ulcers syndrome in the Oti Region of Ghana: Study of cutaneous leishmaniasis, yaws and *Haemophilus ducreyi* cutaneous ulcers. PLoS One. 2023;18(9):e0292034 and Akuffo R, et al. Detection of cutaneous leishmaniasis in three communities of Oti Region, Ghana. PLoS Negl Trop Dis. 2021 May; 15(5): e0009416), we have not seen cases of these 2 skin NTDs in the communities within the study area where we operate skin NTD clinics. We selected the skin NTDs endemic in the study area which are BU and yaws; also, these were the conditions of interest in the larger research project that this research was part of. Our study provides important perspectives on the experiences and effects of stigma among individuals with BU and yaws in central Ghana where the diseases are endemic. The findings from the current study will provide important data that can be incorporated in any systematic review that will be conducted on the subject in the future.

-    Lines 183-192: the team at KCCR was said collaborate with the district health management team at Atwima Mponua “to confirm and manage a yaws outbreak in the district”. However, nowhere in the text is revealed its link to BU cases. How did they get access to the list of patients with BU?

Response: In the results section (Table 2, line 278), we show that all BU cases were recruited from the Wassa Amenfi district only. We further stated in the methods section (line 201-204), that the team at KCCR operates a skin NTDs clinic in the Wassa Amenfi East district and health records were reviewed to identify and trace people who were currently receiving treatment or had been previously treated for BU. Included participants with BU were persons who were attending the skin NTDs clinic operated by the study team in Wassa Amenfi.

-    Lines 196-198: “The sample size was intended to both generate information on geographical variation and disease specific differences between BU and yaws.” This is not accurate, since at any time in the manuscript authors address geographical variations and by “disease specific differences” are the authors talking about differences in stigma? This should be clearer. Additionally, this is an exploratory qualitative study. What is really necessary regarding to population sampling is to include information on how many patients were asked to participate in the study, how many refused, and if geographic variations are relevant, information on the provenience of subjects.

Response: We have revised the section (line 217-218) and it now reads:

‘’The sample size was intended to generate information disease specific differences in stigma between BU and yaws.’’

We also previously stated (line 218-220) that ‘’ All identified individuals affected by either of the two diseases of interest were included to make the sample representative of the experiences of people with BU and yaws in the study area.’’

We have also added to the limitations section the following sentences (line 678-681):

‘’ Differences in disease distribution and reporting practices by health facilities also influenced our recruitment. Most people with yaws came from Atwima Mponua while everyone with BU came from Wassa Amenfi East, but we believe there are enough shared sociocultural characteristics that our analysis is reflective of a broader central Ghanaian social context.’’

-    Lines 217-218: “The guide was pretested in Ahafo Ano North, a nearby district also endemic for BU and yaws.” Is this published? If not, how has it been pretested? Who pretested? Using what population sample?

Response: We have revised the section, and it now reads (line 236-238):

‘’The guide was pretested using mock interviews by the study team on seven affected individuals in Ahafo Ano North, a nearby district also endemic for BU and yaws. No major changes to the study guide were considered necessary.’’

-    Line 228: “Other minor themes were also explored.” Such as? Are the authors talking about the micro experiences in line 230?

Response: We have deleted the sentence from the manuscript.

Reviewer #5: Objectives of the study are identified with a hypothesis being tested.

Samples size: sufficient for this study.

Statistical analysis support conclusions.

Response: Thank you for the commendation

Major revision:

-    The first part of the introduction should be revised, see below.

Response: Done as suggested (line 111-121). See response to reviewer 5 below.

-    The authors should mention how study participation was approved in terms of illiteracies, e.g. thumb print. No further concerns about ethical clearance in general.

Response: We stated in the section on ‘’ethics statement’’ that ‘’Written informed consent was obtained from all participants’’ (line 173).

To address the review comment on consenting for persons who did not understand the English language, we have also added the following sentences to the section (line 174-179):

‘Full disclosure and explanation of the study procedures was provided to all participants in a language of their choice (English or the local language, Twi). Written informed consent was obtained in English from all participants. For individuals who could not read or understand English, consenting was done via an interpreter in the presence of a witness of the individual’s choice. Following this, persons who could not sign, thumb printed their consent for inclusion in the study.’’

Minor revision:

-    “WHO” throughout the document should be spelled as “the WHO”.

Response: Done (line 43 and 71)

Reviewer #4: - The analysis presented matches the overall analysis plan.

- Concerning the presentation of the results and the quality of figures, I suggest the authors to add a summary table of the findings, with one column for BU and another for yaws, including shorter examples of the messages conveyed by participants in each of the dimensions assessed and the percentages of patients that were included in them. The manuscript is lengthy and it would help to better convey the message.

Response: We have presented the results using the standard format for presenting findings from qualitative studies. Indeed, we used the Consolidated criteria for Reporting Qualitative Research (COREQ) checklist. This was attached as Supplementary file S1. Furthermore, the other reviewers agree with the way the results have been presented.

Point-by-point:

-    Lines 238-240: In other studies in BU in African countries, category I/II lesions are the most frequently found. Why are there so many category III lesions in your population when compared to other studies? What is the impact that this has on healthcare seeking and stigma?

Response: Active case search activities are essential for the detection of the early stages of BU. The high proportion of individuals with category III BU may be a reflection of limited case detection activities in the Wassa Amenfi area due to limited financial support over the years preceding the study.

We have previously mentioned the impact of large lesions on stigma in the discussion section (line 585-586) that ‘’ Participants who had non-ulcerative forms of BU reported less exclusion compared to participants who had large ulcers which took longer time to heal.’’ We also mentioned how stigma facilitates health seeking and treatment adherence (line 472-474). We also provided supporting quotes from participants in the results section.

-    Lines 265-270 and 551-560: see the previous point.

Response: See response to reviewer 4 above. Further, we indicated how early-stage BU (non-ulcerated lesions eg nodules are associated with less stigma when we stated that (line 286-287) ‘’ Individuals who had non-ulcerated forms of BU reported less stigma compared to those who had ulcers.’’

-    Lines 426-427: “For participants with yaws, there was no observed effect of stigma on social relationships.” Yet, the authors never discuss hypotheses for this result nor why it could be different from BU.

Response: We have added the following sentences to the discussion section (line 609-615):

‘’We found that for participants with yaws, there was no effect of stigma on social relationships. A possible explanation for this observation might be the fact that yaws lesions tend to be smaller and less disfiguring than BU ulcers and so induce less stigma and be socially acceptable. This is supported by the finding that non-ulcerated forms of BU were associated with less stigma than large ulcers. Additionally, yaws lesions may not have the odorous smell that is sometimes present in large BU lesions especially when they are secondarily infected [47].’’

-    Lines 540-543 need revision:

o    “Further, stigma experiences differ among children and adults.” In BU? In yaws? In both?

o    “Stigma experiences contributed largely to negative outcomes.” Since this is a qualitative study, either this sentence should rephrase to “were perceived in this study to contribute…” or otherwise citations from previous works are needed to back it.

o    “Participants who used religious coping reported higher psychosocial suffering as compared to those who coped using active mechanisms such as confrontation.” There was no formal statistical analysis of this, meaning that this shouldn’t be written so bluntly. An alternative would be “tended to report”.

Response: Thank you for the suggestions. We have edited the section, and it now reads (line 567-571):

‘’In this study, we show that the types and levels of stigma vary for BU and yaws. Further, stigma experiences differ among children and adults in both BU and yaws. Stigma experiences were perceived in this study to contribute to negative outcomes. Participants who used religious coping tended to report higher psychosocial suffering as compared to those who coped using active mechanisms such as confrontation.’’

-    Lines 582-589: it is the only time in the discussion that Goffman theory was mentioned, although it guided the way the work was approached. Shouldn’t this be more discussed?

Response: Goffman’s theory is described in the theoretical framework section of the introduction section (line 151-163). Results are presented as micro and macro level experiences. We also referred to this theory in the discussion (line 620). We do not think further discussion on Goffman’s theory is warranted as doing so will unnecessarily increase the length of the manuscript.

-    Lines 594-596: authors state differences between their observations and previous results regarding the role of religion in managing stigma, but no discussion is provided on the why behind those differences. If comparisons are being made, they should be discussed, even if shortly.

Response: We have done as suggested. The section now reads:

‘’Unlike Indonesia however, affected individuals in the present study did not regard their symptoms as a punishment or test of faith; the observed difference may reflect disease-specific characteristics or the disparities in socio-cultural contexts between Ghana and Indonesia.’’ (line 633-636)

Reviewer #5: Analysis presented per plan. Results are clearly presented, and tables are in order.

Response: Thank you

Reviewer #4: - The conclusions summarize the data presented.

- The limitations of analysis are described.

Response: Thank you

- The way authors discuss how the data can be helpful to advance our understanding of the topic under study does not always match the data presented (please refer to the point-by-point discussion). Furthermore, the omission of relevant references from the literature in the introduction and discussion make it difficult to ascertain the novelty of the findings, which appears to be limited as it is currently presented.

- Public health relevance is addressed, albeit it could be expanded with more concrete actions that could be taken to address the various issues identified. Additionaly, as the authors mention, there were studies on stigma already performed in Ghana. Were there programs estabilished to address stigma after those studies were published? If yes,  could the data the authors now present influence those programs in any way?

Response: No formal programmes to address stigma have yet been integrated into management of NTDs in Ghana. We have included the additional references as suggested. (see response to reviewer 4 above)

-    Lines 646-648: in what way would the authors use the newly acquired knowledge to design and integrate strategies to address stigma?

Response: We addressed this in the conclusion (line 688-695) where we state that ‘’ We propose that the differences in stigma among people affected by BU and yaws, and the role of age in determining types of stigma, should be considered, in the design and implementation of integrated interventions for skin NTDs. Interventions for school health programmes should incorporate teacher education about skin NTDs affecting their communities to avoid misinformation and unnecessary, stigmatizing and deleterious practices such as student exclusion from schools. Teachers are a potentially important human resource in addressing negative impact of stigma on the wellbeing of affected students and promoting the importance of treating affected people with respect.’’

See also response to reviewer 4 below.

-    Lines 651-653: authors mention the role of teachers and their role in addressing the negative impact of stigma. Yet, this was never discussed during the manuscript and the few times that teachers were mentioned in the results section, they were actually part of the problem and not of the solution. So, what is the role of teachers?

Response: We previously stated in the discussion (line 599-602) that ‘’ Individuals with yaws were sent home by school authorities for fear of transferring the disease to other school children; this finding differs from the Philippines where affected children suffered bullying which contributed to school absenteeism [39]. The difference may be related to varying socio-cultural interpretation of disease in the two countries.’’ We have also added the following sentences to the discussion section (line 602-605):

‘’We propose that as part of school health education programmes, teachers should be educated and trained to identify, refer and offer psychosocial support to students affected with skin NTDs.’’

Reviewer #4: -    Lines 201-203: This line should have been in the previous paragraph.

Response: Done as suggested (line 203-204)

-    Line 224: extra blank space in “to ensure”

Response: Done

Reviewer #5: none, besides above mentioned

Response: Thank you.

Reviewer #4: I thank the authors for their manuscript “Stigma experiences, effects and coping among individuals affected by Buruli ulcer and yaws in Ghana”, which focuses on the stigma perceived by individuals afflicted by Buruli ulcer (BU) or yaws. This is a pertinent topic, as previous studies indicated it may by a source of additional mental distress, with potential impact on disease outcomes. To this aim, authors refer to the Goffman’s theory on stigma to conduct a series of interviews with BU and yaws patients, thereby enabling them to identify disparities in the stigma perceived by patients in each of these conditions.

Even though this is the main message of the study and worthy of reflection, there is not a clear alignment between ideas, from the problems raised in the introduction, to the overall objective of the study and the discussion of the results. Furthermore, the manuscript would benefit from a sharper definition of the primary strengths of the approach taken and from a more extended revision of its discussion section, as the potential impact of the findings with more concrete examples is not sufficiently addressed.

I commend the authors for the effort they made in collecting their data and hope to see their manuscript at its best, if not in this journal, in another.

Response: We thank the reviewer for his comments. We have revised the manuscript as per the responses above.

Reviewer #5: The paper is – in general - well written, and analyses seem adequate.

Nevertheless, I would suggest rephrasing the first paragraph of the introduction as this seems to me not adequate for the general reader. It is written too specific and does not allow an insight of the analysed diseases for the reader who is not practiced in these NTD’s.

 Response: We thank the reviewer for the suggestion and have accordingly revised the section. Additionally, we have added the following sentences (line 111-120) to the introduction section to provide additional information on the diseases as suggested by the reviewer:

‘’BU and yaws commonly affect children and are predominant in central and southern Ghana, especially in poor and marginalized communities with limited access to health services, water, and sanitary living conditions [11]. Clinically, BU lesions can present as a nodule, plaque, oedema or ulcer. BU lesions are categorized into 3 groups based on the size as follows: category I- lesions less than 5 cm in diameter, category II- lesions between 5 and 15 cm in diameter and category III- single lesions greater than 15 cm, or multiple or oedematous lesions. Yaws is caused by *Treponema pallidum* subspecies *pertenue* and is characterized by cutaneous lesions (chiefly papilloma or ulcer) during the primary and secondary stages and destructive lesions of the bones in the tertiary stage of the disease [5].’’

Stigma related to NTDs were not yet published on a broad range – publications were limited to a small number of NTDs. Stigmata constitute a large burden on social and economic life of affected patients and other involved persons/communities.

The authors are going to publish a very important article as recommended by the WHO.

Response: We thank the reviewer for the commendation.
