# Supplementary material for: Stigma experiences, effects and coping among individuals affected by Buruli ulcer and yaws in Ghana
Source: PLoS Negl Trop Dis. 2024 Apr 29;18(4):e0012093. doi: 10.1371/journal.pntd.0012093 (PMC11081504; doi:10.1371/journal.pntd.0012093)
Supplement: Attachment — Submitted filename: Response to reviewer comments.docx [file pntd.0012093.s003.docx]

18^th^ January 2024

Kumasi Centre for Collaborative Research

Kwame Nkrumah University of Science and Technology

Kumasi, Ghana

The Editor

PLOS Neglected Tropical Diseases

Dear Sir,

**Re: Decision on your PLOS Neglected Tropical Diseases submission (PNTD-D-23-00867) - [EMID:a8475c733cbba7fe]: ‘Stigma experiences, effects and coping among individuals affected by Buruli ulcer and yaws in Ghana’**

My co-authors and I, wish to thank the editors and reviewers for the feedback on our manuscript.

My co-authors and I have taken note of the review comment and revised the manuscript as suggested. We wish to submit the revised manuscript for publication in your esteemed journal. In the attached reply, the reviewer questions are in blue font and our responses are in red font.

We look forward to the further review of the manuscript and the subsequent publication of same.

We count on your usual kind assurances.

Yours Sincerely,

Dr Yaw Ampem Amoako

Corresponding author

**Point-by-point response to review comments**

Comment: Reviewer #3: The authors have not clearly stated the objectives of the study or clearly articulated research questions derived from their understanding of the problem (either from literature or their observation), which they seek to explore. While stating that this paper will attempts to address gaps, they have identified by exploring stigma experiences of people currently or previously affected by BU and yaws, the authors are requested to be clearer on what they sought to explore in the introduction. Suggestion to organize these objectives/research questions to align with how the results have been presented: experiences of stigma, effects, and coping strategies.

Response: We have revised the section, and it now reads: ‘’We set out to explore the experiences of stigma, effects and coping strategies among individuals affected by Buruli ulcer and yaws in Ghana’’ (Page 4, line 135-137)

Comment: Study design: The qualitative study design using face-to-face interviews employed is appropriate. Although the authors state that they used a purposive sampling technique, they are not clear on the specific characteristics/attributes they based their sampling on. Why then is this not convenience sampling?

Response: We thank the reviewer for the comment. We have revised the section to indicate that convenience sampling technique was used for recruitment of study participants as only persons that were available and willing to participate were included in the study. The section now reads:

‘’This study utilized face-to-face semi-structured interviews. A convenience sampling technique was employed to identify and select participants for the study.’’ (page 6, line 195-196):

Comment: Sample size: More detail is required here. How many cases did they anticipate identifying? How many did they expect to interview? What was the minimum sample size required for meaningful analysis? As this is not a key-informant interview and the authors will be generalizing their findings on patient experiences, this is a significant omission.

Response: We used a qualitative methodology for this study. We intended to include all available individuals with confirmed BU and yaws in the 2 study districts in the 2 years preceding the study to get a first impression of the stigma experiences, effects and coping strategies. The sample size was intended to both generate information on geographical variation and disease specific differences between BU and yaws. Since all identified individuals with the two diseases of interest were included, the sample is representative of experiences of people with BU and yaws in the study area. Furthermore, data saturation in the open questions was included in the decision making on the sample size. We have revised the section and it now reads:

‘’ The sample size was intended to both generate information on geographical variation and disease specific differences between BU and yaws. All identified individuals affected by either of the two diseases of interest were included to make the sample representative of the experiences of people with BU and yaws in the study area. Furthermore, data saturation in the open questions was included in the decision making on the sample size.’’ (page 6, 196-200)

Comment: Methodology: The authors stress the interviews were carried out by female study team members. Is there a special reason why? Is there a possibility of bias limiting the sex of interviewers to one sex?

Response: We thank the reviewer for the observation. Indeed the team that conducted the interviews include a male (MNO); we have revised the section to correct the initial omission and it now reads:

‘’ Between June-July 2022, interviews were conducted by female study team members (RDT & LO) and a male team member (MNO) in private consulting rooms at the health facility during usual clinic visits in the community or at a nearby health facility as convenient for participants.’’ (page 6, line 202-205)

Comment: Ethical/Regulatory concerns: The authors state that health facility records were reviewed to identify and trace people who were currently receiving treatment or had been previously treated for BU or yaws. Who carried out this review? The health facilities have no right to divulge someone’s disease status to a researcher; this is unethical. One suggested approach was for health workers to have obtained some form of consent from identified patients before could be contacted by researchers. If the facilities concerned are Ghana Health Service (GHS) facilities, it would constitute a significant omission on the part of authors not to have sought clearance with the GHS Ethics Research Committee. Was there also regulatory approval by the relevant agency concerned for the health facilities? This is not stated.

Response: Our team at KCCR has collaborated with health facilities in the middle belt of Ghana to provide clinical and laboratory confirmation services for individuals with skin NTDs like Buruli ulcer and yaws. We run a network of skin NTD clinics in the middle belt of Ghana including in the Wassa Amenfi East district. Indeed, our laboratory at KCCR is a key member of the WHO network of laboratories (BULABNET) for the confirmation of Buruli ulcer (Marion E, et al. A combined effort of 11 laboratories in the WHO African region to improve quality of Buruli ulcer PCR diagnosis: The "BU-LABNET". PLoS Negl Trop Dis. 2022 Nov 4;16(11):e0010908. doi: 10.1371/journal.pntd.0010908. PMID: 36331971; PMCID: PMC9668193).

The team has been involved in several key developments on NTDs including the clinical trial that led to the development of a full oral regimen for Buruli ulcer (Phillips RO, et al. Rifampicin and clarithromycin (extended release) versus rifampicin and streptomycin for limited Buruli ulcer lesions: a randomised, open-label, non-inferiority phase 3 trial. Lancet. 2020 Apr 18;395(10232):1259-1267). Our collaboration with the Ghana Health Service and its facilities also includes assisting with the management of infectious disease outbreaks (Amoako YA, et al. A scabies outbreak in the North East Region of Ghana: The necessity for prompt intervention. PLoS Negl Trop Dis. 2020 Dec 22;14(12):e0008902; Ofori-Amoah J, et al. Scabies outbreak investigation and treatment in the Sekyere East District, Ghana: A call to end the neglect. Cogent Medicine. 2021;8(1):1964185). Indeed, the Kumasi Centre for Collaborative Research (KCCR) assisted the district to confirm and manage a yaws outbreak in 2021.

The individuals included in the current study were recruited from the skin NTD clinics which are run by the KCCR team in the study districts. There was no breach of confidentiality regarding disclosure of health records of study participants as disease status was not disclosed by health facilities to researchers. The included participants were individuals whose status were known by the study team as part of usual clinical care at the skin NTD clinics.

All ethics procedures and processes as required by the institutional review boards were followed. The study was approved by appropriate ethics review committees as indicated in the ethics statement. Approval from the Health Facilities Regulatory Agency (HEFRA) is not a pre-requisite for conducting research in Ghana.

We have added the following sentences to the methods section to clarify how patients were contacted for recruitment:

‘’The team at the Kumasi Centre for Collaborative Research in Tropical Medicine (KCCR) runs a clinic for individuals with skin NTDs including Buruli ulcer and yaws in the Wassa Amenfi East district. In 2021, the KCCR collaborated with the district health management team at Atwima Mponua to confirm and manage a yaws outbreak in the district. Individuals who were currently receiving treatment or had been previously treated for BU or yaws from these two settings were contacted for recruitment into the study. Individuals attending the skin NTD clinic at Wassa Amenfi East hospital were contacted in person for recruitment. Participants in Atwima Mponua district were initially contacted via telephone and the study explained to them. Following the initial telephone discussions, an in-person meeting was held with affected individuals during usual clinic visits in the community or at a nearby health facility as convenient for participants. Only individuals who were willing to participate and provide written informed consent were included in the study.’’ (page 6, line 183-194).

Comment: Reviewer #3: Results are clearly presented. Information in tables has been clearly presented.

A minor point is to ensure tables (e.g. Table 2) do not go across pages in the final print.

Response: We thank the reviewer for the comment. This has been corrected.

Comment: Reviewer #3: The conclusions generally support the data presented (while noting earlier concerns about generalizing without adequate sample size and the possibility of bias introduced by limiting sex of interviewers).

The limitations of analysis have also been clearly described.

The authors have also discussed how their findings can advance the understanding of this topic and what public health actions may be required-

Response: We thank the reviewer for the comment. We have addressed the issue regarding the gender of the interviewers (line 202-205). See also response to reviewer 2 below.

Reviewer #2: Ruth Dede Tuwor and co-authors reported stigma experiences, effects and coping among individuals affected by Buruli ulcer and yaws in Ghana.

The study addresses an important psychological aspect in the management of skin NTDs, which is vital to shaping national policies, and thus contributing to skin Health for all.

That notwithstanding, the study has some issues that limit the interest of this paper.

Comment:1-In the introduction, there are some missing data to support the arguments and also the use of repeated statements. In particular the different stigma characteristics and the status reports on the two skin NTDs which are the subject of this work. Speaking of yaws and BU specifically, the studies of Doftas et al (2022, https://doi.org/10.1186/s41182-022-00433-4), Azubuike et al (2023, <https://doi.org/10.1093> /inthealth/ihad090) and Nwafor et al even though cited (doi: 10.4314/ahs.v19i2.34) are guidelines. More broadly, Koschorke et al. (2022, https://doi.org/10.3389/fitd.2022.808955) provide a significant overview.

Response: We have expanded the introduction section to address the reviewer’s comment by adding the following sentences:

‘People with NTDs experience stigma through multiple mechanisms. Biologic factors such as physical impairments caused by NTDs mediate stigma. Furthermore, fear of disease transmission and lack of knowledge about a disease are psychological factors that lead to higher levels of stigmatisation of affected individuals and their families [16]. Different NTDs have their own specific local beliefs linked to stigmatization. Traditional beliefs in witchcraft, curses, or evil spirits have been linked to the stigma experiences in NTDs like leprosy, Buruli ulcer [17], yaws [18] and scabies [19]. In podoconiosis, beliefs about the hereditary nature of the disease are associated with stigma reported by affected individuals [20]. ’’ (page 4, line 117-125)

Comment:2-in the study setting, authors described population, the most common language and ethnics groups in the Atwima Mpouna and Wasa Amenfi East district. But there is no mention of the prevalence of BU and yaws; Authors should add this information

Response: Although there are anecdotal reports of skin NTDs in Atwima Mponua, there has been no formal assessment of the situation in the district. Actual numbers of cases of yaws and BU in the district are unavailable and a formal assessment will be necessary to document the burden of the two diseases in the district.

Comment:3-the authors chose a female team for the interviews. Perceptions/attitudes are sometimes gender dependent depending on cultures. As a result, the creation of an exclusively female team can cause bias in the responses provided. Authors should explain this choice of the investigation team.

Response: We thank the reviewer for the observation. Indeed, the team that conducted the interviews include a male (MNO); we have revised the section to correct the initial omission and it now reads:

‘’ Between June-July 2022, interviews were conducted by female study team members (RDT & LO) and a male team member (MNO) in private consulting rooms at the health facility during usual clinic visits in the community or at a nearby health facility as convenient for participants.’’ (page 6, line 202-205)

Comment:3-There is missing information on the sample size calculation.  Authors should give more explanation on the study sample size considering the disease prevalence, type of lesion age and sexe. The sample size in the study seems to biais the results.

Response: We intended to include all available individuals with confirmed BU and yaws in the 2 study districts in the 2 years preceding the study to get a first impression of the stigma experiences, effects and coping strategies. The sample size was intended to both generate information on geographical variation and disease specific differences between BU and yaws. Since all identified individuals with the two diseases of interest were included, the sample is representative of experiences of people with BU and yaws in the study area. Furthermore, data saturation in the open questions was included in the decision making on the sample size. See also response to reviewer 3 above.

Comment: Reviewer #3: As noted earlier, tables should not go across pages in the final print.

Response: We thank the reviewer for the comment. This will be delt with during the typesetting of the proofs of the accepted manuscript.

Reviewer #1: This is a very interesting study presenting stigma of yaws and BU in rural areas of Ghana and brings to light new and interesting aspects of stigma suffered by those infected with BU and yaws.

Comment: I would address the fact that the number or percentage of participants reporting the different levels of stigma is not included in the results, therefore we dont know if the stigma reported is something only one or most participants reported.

Response: We have revised the section on macro-level stigma as follows:

‘’Community members, school mates and school staff were reported to stigmatize individuals currently or previously affected by BU (n=14, 93.3%). Individuals who had non-ulcerated forms of BU reported less stigma compared to those who had ulcers. The macro-level stigma associated with yaws was reported by 5/16 (31.3%) of affected individuals and their caregivers; this was out of concern of contagion by schoolteachers who considered sending affected children home as a means of protecting other school children.’’ (page 9, line 265-270)

We have also revised the section on micro-level stigma by adding the following sentence (page 11, line 329):

‘’Micro-level stigma was observed for BU (n=15, 100%).’’

Comment: Its interesting that almost no stigma in yaws has been reported, when most studies in yaws report stigma. ( see the study in yaws stigma in Ghana by Marks et al 2017). This should be addressed in the discussion as it is an unexpected outcome.

Response: We previously indicated in the results section that courtesy stigma was present in caregivers and parents of some yaws affected individuals (page 11 line 347). We also indicated that micro-level stigma was common in both BU and yaws in this study, contributing to self-isolation and psychosocial burden. (page 18, line 563-564)

Comment: It would also be beneficial to include a section with the limitations of the study addressing issues such as the small sample size or the sampling methodology.

Response: The study limitations were included at the end of the discussion section. To address the reviewer’s concern, we have included the ‘’study strengths and limitations’’ as a sub-section and expanded it to read as follows:

‘’Our study adds new insight to the stigma literature in terms of its large focus on children and their caregivers, with shared experiences from BU and yaws, bringing and disease-specific differences which are important in informing integrated interventions within the context. The study included all the identified individuals with the disease and so the sample is representative of the experiences of people with BU and yaws in the study area. However, the study was limited in not exploring gender or cultural differences and intersectionality including local beliefs on disease causation and aetiology, which may give further insight to understanding stigma perpetrated against affected individuals.’’ (page 20, line 625-632)

Comment: Reviewer #2: Given the endemicity of the both skin NTDs in the study sites,  sample size constitutes a  bias in drawing conclusions. The type of skin NTD and the phase of the disease (active infection, just healed, & former patient) also constitute a another bias in terms of provided responses. Participants' responses may vary depending on whether the interviewee has the illness, under treatment or is cured. This work requires restructuring to address study design shortcomings. The conclusion of this work should be carried out in subgroup analysis (i.e. sick vs. just recovered, former patient vs. current patient, under treatment, etc.).

Response: Although there are anecdotal reports of skin NTDs in Atwima Mponua, there has been no formal assessment of the situation in the district. Actual numbers of cases of yaws and BU in the district are unavailable and a formal assessment will be necessary to document the burden of the two diseases in the district.

We adopted a qualitative methodology for this study. We had previously included the lesion characteristics of BU and yaws participants in Table 1. Furthermore, throughout the results section, we included quotes from participants with active disease/ current patient (not healed) and those with inactive disease/ former patient (healed) to show that stigma occurs in both categories.

Comment: Based on available data, Ghana bears much of the global yaws burden for example. How can the authors explain so much stigma in a population where a treatable disease is prevalent?

Response: We agree with the observation by the reviewer about the two diseases being treatable. We are also aware that in endemic communities, stigma is usually associated with chronic, difficult to treat and poorly understood conditions. However, within Ghana, stigma has been reported in treatable skin NTDs such as BU, yaws and scabies. We have added the following sentences to the discussion section to highlight these facts:

‘’Stigma and supernatural explanations of disease causation (such as witchcraft and curses) are usually linked to diseases which are difficult to cure or chronic and in which causation is difficult to explain [19]. However, in a previous study in Ghana, some 13% of participants believed that yaws was caused by supernatural forces such as witchcraft, curses or a punishment from god and this belief was not associated with gender, religion or ethnicity [18]. In another study, Buruli ulcer was associated with supernatural causes by persons in endemic communities [17, 52]. The similarity of cultural, religious, ethnic backgrounds and their impact on belief systems may account for the observed findings in the current study but we are, however, uncertain as we did not explore the impact of local beliefs on the causation and aetiology of BU or yaws in this study.’’ (page 19, line 604-613)

Comment: Reviewer #3: A very relevant topic and well written paper. Serious concerns, however, remain with the methodology and ethical/regulatory issues.

Response: We have responded to the review comments as detailed above.
